# Hydrogel Development, Processing and Applications in Agriculture: A Review

**DOI:** 10.3390/gels12030259

**Published:** 2026-03-20

**Authors:** Carmen Mª. Granados-Carrera, Victor M. Perez-Puyana, Mercedes Jiménez-Rosado, Alberto Romero

**Affiliations:** 1 Departamento de Ingeniería Química, Facultad de Química, Universidad de Sevilla, 41012 Sevilla, Spain; 2 Departamento de Ingeniería Química, Escuela Politécnica Superior, Universidad de Sevilla, 41011 Sevilla, Spain; 3 Departamento de Ingeniería y Ciencia de los Materiales y del Transporte, Universidad de Sevilla, 41092 Sevilla, Spain; vperez11@us.es; 4 Grupo de Ingeniería Química, Ambiental y Bioprocesos, Instituto I4, Universidad de León, 24071 León, Spain; mjimr@unileon.es

**Keywords:** circular economy, biopolymers, controlled release, agriculture, fertilizer, biofortification, soil remediation, superabsorbent materials

## Abstract

Hydrogels have emerged as promising functional materials for improving water management and nutrient delivery in agriculture, particularly under conditions of increasing water scarcity and declining soil fertility. However, most commercially available superabsorbent hydrogels are based on petroleum-derived polymers, raising concerns regarding their persistence in soils, potential microplastic formation and long-term environmental impact. In response, significant research efforts are being directed toward the development of biodegradable hydrogels derived from renewable biopolymers. This review provides a critical overview of recent advances in hydrogel systems designed for agricultural applications, with a particular focus on biopolymer-based materials. First, the current landscape of hydrogel technologies used as soil conditioners and controlled-release systems for agrochemicals is contextualized, highlighting the limitations of conventional synthetic hydrogels. Subsequently, the main classes of natural polymers explored for hydrogel fabrication, including polysaccharides (e.g., chitosan, alginate, cellulose and starch) and proteins (e.g., gelatin, keratin and soy protein), are analyzed in terms of raw material sources, gelation mechanisms and structure–property relationships. Their performance in key agricultural functions, such as water retention, controlled nutrient release, soil conditioning and enhancement of plant growth, is also discussed. Finally, the review identifies major challenges that currently hinder large-scale implementation, including mechanical stability, degradation behavior in complex soil environments, nutrient release control and economic scalability. By integrating recent progress and outlining emerging research directions, this work aims to support the rational design of next-generation biodegradable hydrogels capable of contributing to sustainable agriculture and circular bioeconomy strategies.

## 1. Introduction

The world population is expected to reach 10 billion by 2050, increasing global food demand and the yields required from crops, thereby threatening food security [1]. Thus, agricultural activities are the main producers and are essential for the development of sustainable food systems [2,3,4]. However, agriculture is a sector that consumes about 70% of the planet’s freshwater [5]; concomitantly, about 63% of this water is lost through evaporation and runoff [6]. Moreover, climate change is promoting extreme conditions (abiotic stress), including extreme temperatures, drought and an increase in carbon dioxide levels [7]. These changes are particularly detrimental to plants that fail to adapt to environmental shifts, impacting their growth and development [8]. Additionally, the prevalence of pests and diseases is rising, often necessitating an overuse of agrochemical products to maintain crop productivity [9,10,11,12], while trying to maintain safe and sustainable production [13,14].

Conventional fertilizers provide essential nutrients to sustain soil fertility and enhance crop production. However, their efficiency is limited due to rapid leaching in the soil [15,16], which contributes to groundwater contamination, soil pollution and adversely affects human health and the environment [11,17]. Nowadays, there are several conventional fertilizers, such as solid and liquid fertilizers; however, they are required in large amounts, causing damage and water pollution [18,19]. Therefore, there are several approaches to address the direct application of fertilizers, such as regenerative agriculture [20], organic fertilizer [21,22] green manuring [23] or biofertilizers [24,25].

However, nowadays, scientists are focusing on the development of materials that incorporate agrochemical products into a matrix that acts as a vehicle, increasing fertilization efficiency [26,27,28]. Consequently, controlled-release fertilizers (CRFs) are the latest application for bioplastics and hydrogels. Bioplastics are capable of carrying agrochemicals and releasing them in different ways, featuring low-cost and easily industrialized processes [27,29]. They offer the possibility of carrying out biofortification processes, improving the quality of the food produced [30,31,32,33]. However, they possess poor water absorption properties and limited mechanical strength [34,35].

Hydrogels, in contrast, are three-dimensional polymer networks with remarkable water absorption capacity due to hydrophilic groups [36,37,38,39], combined with advantageous characteristics like porosity and swelling features, among others [40,41,42,43]. These materials have multiple applications, such as tissue engineering, dye biosorption and food additives [44,45,46,47]. From an agricultural perspective, hydrogels possess the ability to improve soil properties, facilitate the oxygenation of seeds and plant roots, are environmentally friendly, promote root growth, enhance seed germination and possess tunable mechanical properties [36,48,49,50]. Thus, hydrogels provide a long list of benefits, such as reducing irrigation frequency, decreasing water run-off and acting as a controlled release system [51,52], which makes them stand out [53,54,55,56]. Today, some hydrogels are commercially available, as shown in Table 1, which are primarily based on the utilization of synthetic polymers and exhibit superabsorbent behavior, dominating the market due to the increase in crop productivity and fertility [57]. For example, Control Garden is a brand that fabricates superabsorbent polymers based on potassium polyacrylate and is used for irrigation optimization, enhancing crop development (due to their ability to release nutrients), and acting in adverse conditions [58]. Similarly, Plara synthesized a controlled release of water and nutrient systems using potassium polyacrylate, highlighting these polymers as a defense against soil salinization due to the absence of sodium in their structure [59]. Nonetheless, these systems have a detrimental effect on the environment due to their lack of biodegradability, which impacts soil fertility (i.e., formation of microplastics) and plant health [60,61]. Specifically, these hydrogels are mainly derived from acrylic and acrylamide derivatives, and as reported by Krasnopeeva et al. [57], the residual monomers can alter human health due to the formation of dangerous neurotoxins and have a negative impact on agriculture and the environment due to the accumulation of saturated water in soil. Moreover, their production processes are complex, tending to create expensive products that are not affordable for farmers, necessitating regulatory frameworks that incentivize their use [62].

There is a special interest in the replacement of synthetic polymers with biopolymers, helping in the reduction of waste and providing effective utilization of natural resources [70]. This highlights waste and by-products derived from diverse sources that can be used as raw materials, promoting a more sustainable living environment as well as a circular economy [71,72,73]. Hence, biopolymer-based hydrogels enhance soil organic matter because of their biodegradation, addressing the problem related to the presence of organic matter in soils, which is threatened due to decomposition, deforestation and the uncontrolled use of liquid fertilizers instead of organic fertilizers [74,75]. Similarly, they are capable of mitigating long-term negative impacts on the environment while boosting sustainability, resilience to drought and pests, and productivity [76].

**Figure 1:**
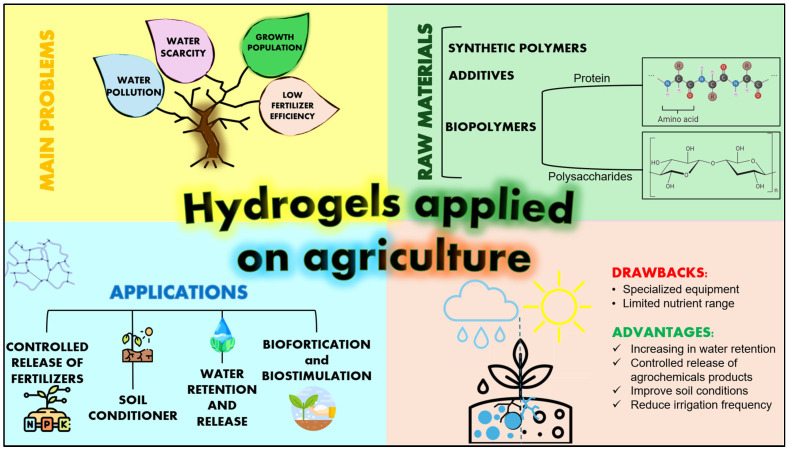
General perspective of hydrogel research in agriculture: main problems, possible raw materials, applications, advantages and drawbacks.

This review aims to contextualize the current state of hydrogel applications in agriculture and identify the key challenges in the development and deployment of these materials. We summarize recent advances in commercial hydrogels, raw materials selection, gelation methods and emerging applications, including some agricultural performance metrics related to these applications. Particularly, biopolymer-based materials are highlighted for their potential to replace non-biodegradable petrochemical products, which are well-established for polysaccharides at the laboratory scale, whereas proteins require extensive evaluation. This features hydrogels as an innovative material in agriculture. An overview of this purpose is depicted in Figure 1.

## 2. Raw Materials for Hydrogels Applied in Agriculture

Hydrogels can be classified according to different criteria, such as their source, crosslinking type or biodegradability. In this case, this review will focus on classification based on their origin, distinguishing between synthetic polymers and natural polymers (also referred to as biopolymers) (Table 2) [76,77], whose major advantages and disadvantages are displayed in Table 3.

### 2.1. Synthetic Polymers

Synthetic polymers derive from petrochemicals and possess durability, gel strength and absorption capacity [57,78,79]. However, their disadvantages include their degradation rate, potential toxicity, and the possibility of the formation of microplastics after their degradation [80,81,82].

Among the synthetic polymers used in soils as hydrogels for the enhancement of their physical properties, polyacrylamide, polyacrylates and their derivatives are the most prevalent. These polymers exhibit a high degree of swelling in water due to the presence of hydrophilic groups that facilitate water absorption. The most commercially available superabsorbent hydrogels are based on acrylic acid (AA) (Table 1), a synthetic monomer that enables the controlled release of agrochemical compounds while also providing excellent mechanical properties [83]. Regarding their fabrication, hydrogels can be prepared via chemical crosslinking, typically using ammonium persulfate as an initiator and, subsequently, different crosslinkers (for instance, N, N’-methylenebis(acrylamide) (NMBA), Fe^3+^ ions) [84,85] or via irradiation-induced crosslinking methods such as gamma irradiation [86,87]. However, these reagents are expensive and generate undesired residues, which are toxic to soils and plants [84,85]. A solution is their combination with other polymers such as carboxymethyl cellulose or acrylamide [84,86,88,89,90,91,92].

On the other hand, acrylamide (AM), one of the most widely employed polymers for the development of smart hydrogels, can be obtained via chemical modifications, employing crosslinkers (such as NMBA, ethylene glycol dimethacrylate), or via irradiation-induced crosslinking modifications such as gamma irradiation [85,93]. Among its derivatives, polyacrylamide (PAM) is traditionally used as a soil conditioner by enhancing the water retention capacity of soils because of the concatenation of acrylamide monomers [91,92,94,95,96,97,98], while methacrylic acid (MAA) forms pH-sensitive hydrogels with a high degree of swelling [99,100,101,102,103,104].

Moreover, polyvinyl alcohol (PVA) and polyvinylpyrrolidone (PVP) are synthetic polymers that form hydrogels through diverse physical or chemical crosslinking methods, including freeze–thaw cycles, irradiation and the utilization of different crosslinkers, such as glutaraldehyde, formaldehyde and their derivatives [105]. Specifically, these polymers are extensively employed as a substrate for promoting plant growth due to their low toxicity, mechanical stability and high water absorption [106,107,108,109,110,111,112].

Other alternative polymers include hydrophilic polyurethane (PU) and polyethylene glycol (PEG), which exhibit hydrophilicity and excellent mechanical properties. However, PU can be toxic to human health and the environment, while PEG is non-hazardous. Thus, current research focuses on optimizing PEG gelation processes to develop hydrogel materials with potential applications in water retention capacity [113,114,115].

### 2.2. Natural Polymers

Natural polymers (biopolymers) are derived from renewable sources and possess several desirable attributes, including biodegradability, abundance, and affordable price. These properties render them an excellent candidate for hydrogel fabrication (Table 4 and Table 5) while promoting the sustainable utilization of natural resources. However, researchers are attempting to develop new approaches to optimize their limited stability and mechanical properties [76,116,117]. They can be divided into polysaccharides and proteins, as described above.

#### 2.2.1. Polysaccharides

Polysaccharides comprise a diverse array of monosaccharide units linked by glycosidic bonds and are particularly well-suited for agricultural purposes due to their hydrophilicity, availability and low cost [118,119]. Therefore, they are more commonly employed in CRFs and to reduce irrigation frequency, owing to their stability and the high water affinity of their hydrophilic functional groups [76,120]. Nevertheless, several challenges remain to be overcome to optimize their capacity to manufacture superabsorbent materials.

##### Chitin

Chitin is a polysaccharide that is the second most abundant biopolymer in nature. It is sourced from a diverse range of organisms, including crustaceans, insects and invertebrate animals [121,122]. Several studies have focused on the development of hydrogels based on this biopolymer, which can exhibit biodegradability, non-toxicity, as well as antibacterial and antiviral properties [121,123,124]. The results demonstrate that chitin is highly effective for use in soil culture media, facilitating the germination and growth of plants [125,126]. Moreover, incorporating nitrogen into soils due to its biodegradation is essential for these processes [126].

**Table 4:** Characteristics of different biopolymers used (polysaccharides and proteins) for the development of hydrogels in agriculture.

	Biopolymers	Characteristics	References
**Polysaccharides**	Chitin	Non-toxicity Antibacterial and antiviral properties	[121,122,125,126,127]
	Chitosan	Easily soluble in several organic acids Antiviral, antibacterial and antifungal properties Non-toxicity Gelation methods: chemical crosslinkers (glutaraldehyde, epichlorohydrin), ionic compounds (lithium chloride) and pH change	[121,123,124,128,129,130,131,132,133,134,135,136,137,138,139,140,141,142,143,144,145]
	Sodium alginate	Poor mechanical properties at hydrated state Enhancement in mechanical properties with divalent ions (i.e., Ca^2+^) Distinctive water absorption capacity, allowing superabsorbent materials	[76,109,146,147,148,149,150,151,152,153,154,155,156,157,158,159,160,161,162,163]
	Agar	Mainly, combined with synthetic polymers (AA, MA …) Renewability Adequate response under drought conditions	[112,164,165,166,167,168,169,170]
	K-carrageenan	Obtention from red algae Gelation methods: cooling or presence of metallic ions	[171,172,173,174,175]
	Starch	Abundance in plants Inexpensive Non-toxic Good water retention Gelation in 3 steps: (1) hydration, (2) destruction of granules, (3) cooling Crosslinking with citric acid	[76,137,176,177,178,179,180,181,182,183,184]
	Cellulose	Most abundant polysaccharide Presence of hydroxyl groups in its structure, allowing a hydrophilic nature Gelation methods: chemical crosslinkers (epichlorohydrin, citric acid or iron (III) chloride hexahydrate) Possibility of employing environmentally friendly crosslinkers	[54,76,171,173,185,186,187,188,189,190,191,192,193,194,195,196]
	Lignin	High thermal stability Non-toxicity Ease of availability	[82,151,197,198]
	Xanthan gum	Gelation: chemical reagents (citric acid, sodium trimetaphosphate, tannic acid)	[199,200,201,202,203,204,205]
	Guar gum	Hydrophilic nature Benignity	[206,207,208,209]
**Proteins**	Collagen	Poor mechanical properties Resistance to enzymatic degradation Source of organic nitrogen for improvement in plant germination	[118,210,211,212]
	Gelatin	Affordability	[140,146,213,214,215]
	Keratin	Mechanical stability	[216,217,218]
	Soy protein	Formation of superabsorbent materials due to the presence of aspartic and glutamic acids in its structure	[219,220,221]

**Table 5:** Applications of different biopolymers used (polysaccharides and proteins) for the development of hydrogels in agriculture.

	Biopolymers	Application	References
**Polysaccharides**	Chitin	Plant growth regulator, promoting seed germination Nitrogen source in soils through biodegradation	[121,122,125,126,127]
	Chitosan	Plant nutrients Water reservoir CRF Antimicrobial properties Enhancement of the diffusion coefficient of fertilizers	[121,123,124,128,129,130,131,132,133,134,135,136,137,138,139,140,141,142,143,144,145]
	Sodium alginate	Soil conditioner, reducing potassium and nitrogen ions CRF through electrostatic interactions	[76,109,146,147,148,149,150,151,152,153,154,155,156,157,158,159,160,161,162,163]
	Agar	Water reservoir Enhances microbial degradation Promotion of crop growth	[112,164,165,166,167,168,169,170]
	K-carrageenan	Plant stimulant, improving plant productivity and root development Water reservoir Metallic cations retainer Encapsulation and controlled release of bioactive substances	[171,172,173,174,175]
	Starch	Active agent carrier Soil conditioner	[76,137,176,177,178,179,180,181,182,183,184]
	Cellulose	Nutrient-controlled release Thermal stability Soil water retention	[54,76,171,173,185,186,187,188,189,190,191,192,193,194,195,196]
	Lignin	Extends soil water retention, particularly in arid zones Enhancement of the photosynthetic capacity of plants	[82,151,197,198]
	Xanthan gum	CRF Soil conditioner Biostimulant	[199,200,201,202,203,204,205]
	Guar gum	Water reservoir Soil conditioner	[206,207,208,209]
**Proteins**	Collagen	Water reservoir CRF	[118,210,211,212]
	Gelatin	Improvement in shoot and root length under extreme conditions Water reservoir Nitrogen management	[140,146,213,214,215]
	Keratin	CRF Improvements in plant growth	[216,217,218]
	Soy protein	Improvements in plant growth CRF Water reservoir	[219,220,221]

##### Chitosan

Chitosan is a polysaccharide derived from chitin, obtained through partial deacetylation (66–95%) of the aforementioned polymer. In particular, the degree of deacetylation is an essential characteristic, as it is intimately associated with the functionality of the biopolymer. Moreover, chitosan is easily soluble in several organic acids, such as acetic acid and formic acid, among others [121,124]. Its antiviral, antibacterial or antifungal properties, along with its renewability, biodegradability and non-toxicity, make it an essential component in soil fertilizers, particularly in the case of low molecular weight chitosan [121,123,124]. The production of hydrogels using chitosan can be achieved through different approaches: (i) the use of chemical crosslinkers such as glutaraldehyde [136] or epichlorohydrin [125], (ii) the incorporation of ionic compounds such as lithium chloride [126], and (iii) the application of a pH change using NaOH [142].

Thus, this biopolymer has been widely used, and its use in hydrogels promotes the controlled release of fertilizers. For instance, Suratman et al. (2020) [136] and Ladeira et al. (2021) [138] developed hydrogels based on chitosan and carboxymethyl chitosan, which release 30% of NPK in 24 h. Similarly, Yuan et al. (2023) [140] synthesized hydrogels based on gelatin, chitosan and polylactic acid, which exhibited enhanced diffusion in comparison with pure urea, with a diffusion coefficient 1350 times longer.

##### Sodium Alginate

Sodium alginate is a polysaccharide derived from algae and bacteria and is a significant biopolymer used in agricultural applications. This biopolymer is notable for its properties, including non-toxicity, biodegradability and sustainability [76,161,222]. Specifically, alginate can be crosslinked by incorporating Ca^2+^. However, the utilization of this biopolymer is limited by several disadvantages, including its poor mechanical properties, particularly when the hydrogels are hydrated [161,162]. Most scientists have concentrated their efforts on the development of hydrogels using divalent ions, such as calcium instead of magnesium. This is due to the enhancement in mechanical properties that occurs when calcium ions are incorporated [162,163,223]. For example, previous studies have demonstrated that alginate-based hydrogels can be effectively employed in agricultural soils due to their rapid water absorption and ability to gradually release fertilizers by reducing potassium and nitrogen ions [109,160,224]. Similarly, Lv et al. (2024) [157] developed hydrogels by combining sodium alginate with starch, which enhanced the controlled release of urea by incorporating –OH groups that interact with urea and create electrostatic interactions with the soil. In addition, this type of hydrogel also exhibits another distinctive property, namely its capability to absorb water, which enables the production of superabsorbent materials [225]. In this sense, Song et al. (2020) [151] synthesized hydrogels by combining sodium alginate and konjac flour, demonstrating how these renewable hydrogels promoted vigorous growth in tomato plants, extending the growth cycle of these plants by 9–14 days under drought conditions.

##### Agar

Agar is a renewable resource derived from algae (mainly red and brown varieties). It can dissolve in water at temperatures around 83–85 °C. Specifically, this sulfated polysaccharide has been extensively employed as a gelling, thickening and stabilizing agent in the food industry [170,226]. However, this biopolymer has also been employed as a water reservoir in agriculture due to its biodegradability, non-toxicity, controlled release of agrochemicals and responsiveness to microbial degradation [112]. In this instance, this polysaccharide has been extensively used in conjunction with synthetic polymers such as acrylic acid, methacrylamide or methyl acrylate, using gelation methods based on free radical copolymerization with N, N-methylenebis(acrylamide) (crosslinker) and ammonium persulfate (initiator) [112,165]. However, to avoid the implications of non-renewable polymers, scientists such as Dissanayake et al. (2024) [166] have developed 3D-printed hydrogels that can release urea in a controlled manner for 72 h. Another alternative for enhancing their properties is blending with other biopolymers, such as sodium alginate, as reported by Singh et al. (2025) [227] for the controlled release of glyphosate herbicide. For instance, Merino et al. (2023) [169] combined agar with limonene and orange peel to produce a biopesticide, allowing for up to 5 cycles of swelling-deswelling and acting as a water source for tomato plants under drought conditions.

##### K-Carrageenan

K-carrageenan is a linear anionic polysaccharide extracted from red algae. It has been widely used as a plant stimulant, increasing plant productivity and root development [173,174,203]. The gelation of this biopolymer can be facilitated by cooling conditions or the presence of metallic ions, which are generally incorporated with other biopolymers, producing hybrid hydrogels [175]. Other applications include the encapsulation and release of bioactive substances. For example, Pandya et al. (2024) [172] developed chitosan-based hydrogels incorporating k-carrageenan, which exhibit a controlled release of urea, acting as a promising vehicle for agrochemical products as well as a soil conditioner. Similarly, Akalin et al. (2020) [173] elaborated hydrogels based on carboxymethyl cellulose and K-carrageenan, allowing a controlled release of zinc and showing good performance in wheatgrass growth. These results are related to the existence of carbohydrate molecules, comprising carbon, hydrogen and oxygen, which can be used for energy generation and incorporated into plant growth.

##### Starch

Starch is a polysaccharide whose structure is composed of a combination of amylose and amylopectin, which can be described as linear and non-linear forms of starch, respectively. This carbohydrate is abundant, inexpensive, non-toxic, and biodegradable, enabling the formation of hydrogels in agriculture that exhibit beneficial properties (such as good water retention and biodegradability) [76,182,228]. This compound is abundant in plants such as corn, rice, and potatoes, and its gelation can be developed through three relatively simple steps. The first step is initial hydration, during which the granules of starch absorb large amounts of water. The second step is the destruction of the granules as a result of their dissolution in water. Finally, cooling is performed to complete the gelation process [182,183,184,229]. There are studies in which this biopolymer has been combined with synthetic polymers such as acrylic acid or acrylamide through graft polymerization [110,181]. These studies have demonstrated that the incorporation of small quantities of acrylamide enhances the swelling ratio of the hydrogels (the highest value is 780 g/g), facilitating their utilization as water reservoirs in arid conditions and the development of pH-sensitive systems for the controlled delivery of agrochemical products [177]. Similarly, Gungula et al. (2021) [179] developed a starch-based hydrogel using borax as a crosslinking agent, demonstrating excellent water absorption and retention capacities and a controlled release of urea for 37.5 days. On the other hand, alternative methods for the formation of starchy hydrogels exist, such as chemical crosslinking using citric acid, which facilitates the controlled release of potassium and nitrogen [178].

##### Cellulose and Its Derivatives

Cellulose is one of the most abundant polysaccharides on Earth, and it can be found in cotton, wood, and numerous other plants. Specifically, this biopolymer is distinguished by the presence of multiple hydroxyl groups in its structure, which exhibit hydrophilic behavior, allowing for the absorption and retention of significant quantities of water [76,186,230]. Hydrogels can be prepared through physical crosslinking (hydrogen bonding) [231] or through chemical crosslinking, for example, using epichlorohydrin [193,194], citric acid [54,185,190,191] or iron (III) chloride hexahydrate [173,196]. Cellulose is a subject of considerable research interest, although it is possible to find studies that utilize cellulose derivatives due to its biocompatibility, the possibility of formation of hydrogels through physical or chemical crosslinking, its ability to disperse in water, and the possibility of using environmentally friendly crosslinkers [232,233,234,235]. One notable example is the study conducted by Shang et al. (2023) [189], in which carboxymethyl cellulose/acrylamide-based hydrogels were developed. These hydrogels exhibited excellent temperature sensitivity and swelling capability (up to 2056%), releasing 98% of the urea loaded over 6 h. On the other hand, Ahmad et al. (2023) [194] synthesized a controlled-release fertilizer using cellulose as a matrix and epichlorohydrin as a crosslinker, exhibiting a high swelling capacity, rendering it suitable for use in soil water retention. Moreover, another potential alternative for enhancing the mechanical properties of these hydrogels is through the combination with other biopolymers. This approach was exemplified in the research conducted by Charoenchaitrakool et al. (2024) [188], who synthesized hydrogels by blending carboxymethyl cellulose and gelatin, using glutaraldehyde as a crosslinker. This hydrogel demonstrated a promising application due to its sustainable release of urea over 17 cycles (considering a cycle as the procedure that consists of spilling an amount of 50 mL of deionized water on the hydrogel charged with urea and measuring the amount of urea released in the filtrate dissolution), optimizing urea retention. Finally, other studies have concentrated on the development of hydrogels using non-toxic and eco-friendly crosslinkers, such as citric acid. This approach was employed by Durpekova et al. (2020) [236] due to the ability of citric acid to generate hydrogels with the necessary rigidity to maintain their shape and a high swelling ratio.

##### Lignin

Lignin is the second most abundant biopolymer, following cellulose, and it provides rigidity and mechanical strength to plants, facilitating the transportation of nutrients. Its advantages include high thermal stability, biodegradability, non-toxicity, biocompatibility and ease of availability [82,151,198]. Therefore, this compound has been employed in agricultural applications as a soil conditioner in arid zones. The development of lignin-based hydrogels using polyethylene glycol (PEG) as a crosslinker has demonstrated good swelling capacity, although it is lower than that of commercial acrylate-based hydrogels (34 g/g vs. 100 g/g) [197]. Moreover, most lignin-based hydrogels are produced through the blending of biopolymers, such as sodium alginate [151], which have been demonstrated to enhance the photosynthetic capacity of plants under drought conditions.

##### Xanthan Gum

Xanthan gum is a water-soluble, anionic and microbial polysaccharide widely used in plant regeneration due to its bio-stimulatory behavior [203]. The gelation process can be developed using different chemical agents, such as citric acid, sodium tri-metaphosphate (STMP) or tannic acid. These substances promote the formation of an esterification reaction between the crosslinker and the hydroxyl groups present in the xanthan gum structure [204,205]. The potential of xanthan gum-based hydrogels as soil conditioners for promoting plant growth has been previously investigated by Sorze et al. (2023) [201], who combined xanthan gum with cellulose fibers to develop hydrogels with enhanced water absorption and superior performance compared to commercial ones. On the other hand, Das et al. (2023) [202] developed hydrogels based on gelatin and xanthan gum using a cooling process that promoted a controlled release of urea for 32.5 days and demonstrated the ability to absorb high amounts of water after 5 cycles of reswelling. Moreover, these hydrogels were proposed as effective soil conditioners due to the results in the growth measurements of Okra plants.

##### Guar Gum

Guar gum is a non-ionic polymer with a high molecular weight extracted from the seeds of the guar plant, which belongs to the Leguminosae family. The hydrophilic nature, benignity, biocompatibility and biodegradability of this inexpensive polymer are among its most notable properties [208,209]. Polyethylene glycol (PEG) has been demonstrated to be an effective agent for the polymerization of guar gum-based hydrogels in combination with methyl methacrylate (MMA). This approach has been employed by Songara et al. (2021) [206] to create superabsorbent hydrogels with high swelling capabilities (52–110 g/g), which have been shown to confer drought tolerance to plants. Furthermore, the combination of guar gum with synthetic polymers is also possible, such as acrylic acid (as sought by Thombare et al. (2018)) [207], using ethylene glycol dimethacrylic acid (EGDMA) as a crosslinking agent to form water retention systems due to the high values for the swelling degree (470.2–806.2 g/g, depending on the solvent used).

#### 2.2.2. Proteins

Proteins are a wide range of biological molecules that provide amino acids and bioactive peptides, and they have potential as alternatives to conventional petroleum-based synthetic polymers, being safe for humans and environmentally friendly [237]. In comparison to polysaccharides, their chemical structure is composed of numerous functional groups, which makes them more sensitive to external changes and capable of chemical modification [238].

Particularly, proteins can be mainly derived from animals and plants, highlighting the preference for plant-based products due to people’s concerns about health and diets as well as their potential alignment with sustainable sources not derived from animal ingredients [239]. On the one hand, animal proteins include different products such as collagen, gelatin, keratin, etc., whereas plant proteins include protein derived from zein, soy or wheat gluten, among others [240].

Generally, the gelling of animal proteins is higher than that of plant proteins; however, acting parameters such as pH, ionic strength and protein concentration affect the gelation properties [241]. Nevertheless, plant-derived proteins possess several advantages, such as low cost and the possibility of being obtained from available biomass, such as residues and by-products derived from agri-food waste [242]. Their gelation processes are mainly affected by the unfolding of the native protein [238], which can be achieved through relatively simple methods, including hydrophobic or electrostatic interactions, hydrogen bonds or heat treatment (Section 4) [51,116]. However, in general, these biopolymers are often combined with synthetic polymers or other biopolymers to form hybrid hydrogels [51]. Moreover, the use of proteins can enhance plant growth due to the presence of nitrogen in their structure and facilitate photosynthesis [243]. Nevertheless, protein-based hydrogels for agricultural applications are not widely explored, presenting opportunities for investigation [220].

##### Collagen (Gelatin)

Collagen is a protein that is naturally obtained from bone and animal skin. It displays poor mechanical properties and resistance to enzymatic degradation. Nevertheless, this element has been employed in several fields, such as tissue engineering, wound healing or food membranes [211,212,244]. Nowadays, there is a growing interest in the application of this compound for soil remediation purposes, particularly as a source of organic nitrogen, to enhance plant germination [212]. On the other hand, gelatin is a natural polymer derived from collagen that has also been widely used in the wound healing or cosmetic industries [76]. It possesses efficacious characteristics, such as biodegradability, affordability, and the capacity to form gels with ease [213].

Several studies involve the utilization of these compounds. For instance, Tzoumani et al. (2019) [210] developed hydrogels combining collagen with different polyacrylic acid derivatives, which demonstrated a controlled release of fertilizer.

Furthermore, an alternative method for the formation of hybrid hydrogels involves the blending of components such as polysaccharides with proteins. This approach was employed by El-diehy et al. (2024) [214], who researched the development of sodium alginate/gelatin-based hydrogels combined with polyacrylamide and produced by gamma irradiation. Specifically, these hydrogels exhibit enhanced swelling properties and are capable of retaining water for up to 36 days. Similarly, López-Velázquez et al. (2019) [215] developed hydrogels based on gelatin, starch and poly (vinyl alcohol), using a pH change followed by a decrease in temperature for the development of the gelation procedure, enhancing the water uptake capacity (10.8–12 g/g) and optimizing the biodegradation rates after 28 days.

##### Keratin

Keratin is a protein with a high percentage of amino acids that promotes numerous advantages, such as biocompatibility, biodegradability and mechanical stability. Thus, Wattie et al. (2018) [218] evaluated the properties of acrylic acid/keratin-based hydrogels obtained by graft polymerization, demonstrating their high swelling capacity in distilled water (pH = 7), with values between 335.47 and 501.58 g/g. Moreover, some works support the existence of hydrogels sensitive to external stimuli [216]. For example, two studies evaluated the optimization of the blending conditions of acrylic acid/keratin mixtures to obtain hydrogels with high swelling capacity. One study reported a swelling capacity of 1430.7% [216], while another reported a value of 666.67 g/g [217]. In both cases, the same initiator (ammonium persulfate) and the same crosslinker (NMBA) were used.

##### Soy Protein

Soy protein is a plant protein that contains a high proportion of aspartic and glutamic acids, which facilitates the formation of hydrogen bonds and enables the creation of superabsorbent materials [245]. This raw material has been widely used for the formation of bioplastic matrices for the controlled release of zinc [246]. However, there are only a few studies that have evaluated its use for the formation of hydrogels, with different gelation processes, such as thermal treatment [247] or the use of divalent ions [248]. Ardra Ashok et al. (2024) [220] conducted a study evaluating the behavior of soy protein as a hydrogel, developing polyvinyl alcohol (PVA) and soy protein isolate (SPI) hydrogels using citric acid as both a crosslinker and a structuring agent. These hydrogels were able to control the release of urea over 28 days, with 74.1% of the total urea amount released.

### 2.3. Additives

**Figure 2:**
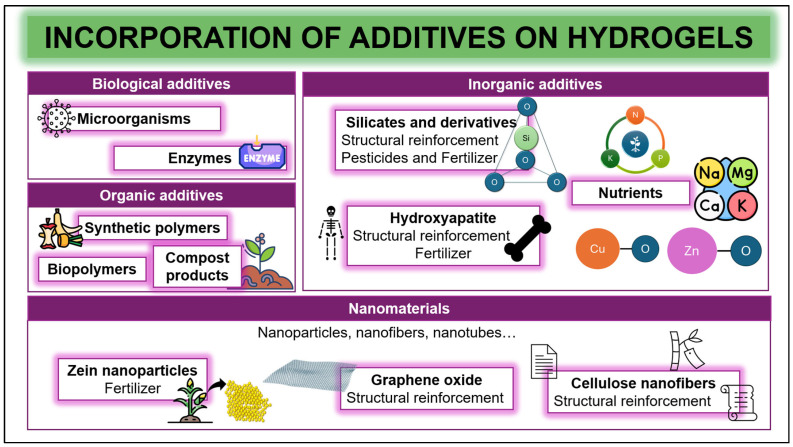
Overview of different additives for hydrogel modification in agriculture, classified as biological, nanomaterials, and inorganic and organic additives.

A variety of additives (Figure 2) is available, depending on different classifications. Particularly, in this review article, 4 sections are distinguished, including organic and inorganic compounds, biological additives and nanomaterials.

#### 2.3.1. Organic Additives

Firstly, the aforementioned synthetic and natural polymers can be employed as additives in the formulation, as well as other polymers such as PVA or PEG. However, interesting organic compounds include humic substances as well as compost. On the one hand, compost has been used for years as an organic amendment due to its potential to enhance soil properties while serving as organic matter. There is special interest in its combination with hydrogels using the compounds formed through the composting process [249]. Hence, one of the most novel materials added to hydrogels is humic substances, which are heterogeneous high-molecular-weight compounds formed during the composting of different residues, presenting a large number of functional groups that endow these substances with multiple characteristics (acid–base properties, amphiphilic nature, etc.) that are essential for the regulation of plant growth. As the majority of these functional groups are free in aqueous media, hydrogels can interact with these types of substances, participating in bonds and acting as natural pesticides while improving nutrient preservation or tolerance to drought stress [250,251]. For instance, Zeng et al. (2025) [252] modified gelatin hydrogels with the incorporation of humic substances, showing an increase in the swelling percentage of the material and maintaining moisture levels.

#### 2.3.2. Inorganic Additives

Inorganic additives include a wide variety of materials such as minerals, zeolite, biochar, silicates, etc. Firstly, one alternative is hydroxyapatite, a phosphorus-containing mineral that is biocompatible, non-polluting and environmentally friendly. The primary investigations aim to develop a material for the controlled release of phosphorus, achieved through the slow release of PO_4_^3−^, which enriches soil conditions and promotes plant growth, thereby avoiding eutrophication [253]. For example, Nooeaid et al. (2024) [254] developed materials based on alginate and incorporating hydroxyapatite, demonstrating an enhancement in plant growth.

On the other hand, alternative options include silicates and their derivatives. Silica, characterized by a tetrahedral structure, biocompatibility, high melting point, and good adsorption properties, is used in Portland cement, as an additive in food production and preservation, hydraulics, and pharmaceutical packaging [255]. In the agricultural field, silica modifies the physical, mechanical, and swelling characteristics of materials by introducing hydrophilic hydroxyl groups that can interact with other groups, forming physically crosslinked hydrogels and thereby reinforcing the mechanical strength of the hydrogel [256]. For instance, Ren et al. (2023) [257] modified a copper-alginate/chitosan hybrid hydrogel to use silica as a reinforcement for a soil fumigant that encapsulates fertilizer, improving the water-holding and retention capacity of the systems and helping in plant disease management and the maintenance of soil water conditions. Similarly, Hafezi et al. (2023) [258] developed a slow-release hydrogel that incorporated silicate derivatives (nano-clay) to enhance strength, resistance to chemical environments and long-term durability while reducing degradation rates.

Zeolite, a non-toxic crystalline aluminum silicate, has the capacity to promote the controlled release of agrochemical products due to an increase in the interactions between the hydrogel and the fertilizer [259,260]. For instance, Tanaka et al. (2023) [261] developed a hydrogel combining zeolite with chitosan or methylcellulose, promoting swelling values between 2.6 and 4.4 g/g in acidic pH while limiting fertilizer release. It is also possible for it to be incorporated into synthetic polymer-based hydrogels, such as the one synthesized by Songara et al. (2022) [262], who combined guar gum and acrylic acid, elaborating a hydrogel that could be used for soil conditioners, nutrient delivery vehicles and water reservoirs.

Moreover, addressing challenges such as climate change and increasing food demand, especially in developing countries, requires the incorporation of essential nutrients into soils [51]. In this sense, nitrogen is one of the principal and depleted nutrients in agricultural soils. It is typically supplied in the form of urea, which contains around 46 wt.% of nitrogen and is relatively cost-effective [16,263]. However, less than 50% of the applied nitrogen is effective due to surface runoff, leaching or vaporization, contributing to global warming and climate change [16,263,264,265]. Other essential nutrients include phosphorus, a critical component for plant growth. However, together with nitrogen, they can cause eutrophication, which not only results in environmental pollution but also poses health risks [16,264,265,266]. Thus, scientists have developed many researchers who have incorporated this nutrient, increasing the efficiency of controlled release of agrochemicals in a range of 6–30 days [159,193,267]. Other alternative options are hybrid fertilizers, which combine the elements with a limited number of secondary nutrients, such as calcium (Ca) and magnesium (Mg), among others [268].

On the other hand, micronutrients are essential for several plant metabolic reactions (including amino acid production or photosynthesis). Hence, some studies focus on the utilization of copper oxide and zinc oxide nanoparticles as nano-fertilizers, which can be easily absorbed by plants and provide controlled release of these elements in water and soil [269]. For example, Shang et al. (2021) [270] enhanced nutrient supply and lettuce plant growth by incorporating copper oxide into hydrogels while mitigating the environmental and health risks associated with high concentrations through controlled release.

#### 2.3.3. Nanomaterials

Nanomaterials include nanoparticles, nanofibers and nanotubes, which are materials that can promote an enhancement in agricultural hydrogels through different crosslinking strategies and the incorporation of different nutrients [43,51,271]. However, due to the mainly hydrophobic nature of many nanomaterials, their incorporation into hydrogels for agricultural applications has not yet been extensively explored [43,271].

Although hydrogels have many attractive properties, their mechanical performance can be improved through various reinforcement methods, such as incorporating strong fibers and nanoparticles into the matrix [54,272]. Specifically, cellulose nanofibers are a promising material due to their nanometer diameter and increase specific surface area, exhibiting a high absorption capacity (an essential characteristic for water retention in agriculture) [193]. In fact, several studies incorporate different reinforcing materials, like those by Das et al. (2023) [54], who prepared cellulose-based hydrogels reinforced with nanofibers to improve mechanical strength and maintain soil vitality. Liu et al. (2021) [109] developed hydrogels based on sodium alginate, polyvinyl alcohol and cellulose nanofibers, significantly modifying the mechanical properties of the materials while allowing the controlled release of agrochemical compounds.

Graphene and other derivative compounds are highlighted due to their exceptional mechanical strength. Moreover, stabilization on the surface of the hydrogel is achieved for the controlled release of fertilizers and nutrients by incorporating these materials [114]. For instance, Liu et al. (2017) [273] elaborated on graphene oxide/cellulose-based hydrogels that possess both high thermal stability and good adsorption properties. In this line, Azeem et al. (2023) [114] combined guar gum and PEG, incorporating graphene oxide, forming a hydrogel that displays an enhancement in water retention, sustained boron release over 30 days and biodegradability.

However, there are other improvements in incorporating nanoparticles for crop protection, specifically through the nanoencapsulation of soil compounds like zein, which is a prolamin protein extracted from maize [274,275]. Specifically, the incorporation of these compounds provides active agents that possess a repellent activity, as shown by de Oliveira et al. (2020) [276], who prepared cellulose derivative-based hydrogels with zein nanoparticles, showing high repellent activity against the major agricultural pests.

#### 2.3.4. Biological Additives

Finally, a recent trend involves the encapsulation or immobilization of microorganisms (including bacteria, fungi, among others) into hydrogel systems to achieve successful plant growth, protecting them against pests and pathogens while increasing soil quality because of their ability to secrete phytohormones that increase nutrient availability, phosphate solubility and nitrogen fixation. Moreover, at the same time, the hydrogel acts as a capsule that protects microorganisms against abiotic stress, thereby improving the moisture retention capacity of inoculants and serving as a biofertilizer [277,278]. For instance, Lima-Tenório et al. (2024) [279] encapsulated bacteria into synthetic-based hydrogels combined with gum arabic, which protected plants from adverse soil conditions while enhancing the fresh weight of maize shoots and roots due to a better associative relationship between the plant and bacteria. Similarly, Valle et al. (2025) [280] developed carboxymethylcellulose-based hydrogels that incorporated bacteria and provided safer storage of inoculants, achieving the supply of S while maintaining cell viability.

These innovative approaches not only demonstrate the versatility of hydrogels in sustainable agriculture but also open new avenues for research focused on optimizing microbial encapsulation for enhanced soil health, nutrient cycling and crop productivity.

## 3. Gelation Process of Hydrogels: Mechanisms and Stimuli for Gelation

**Figure 3:**
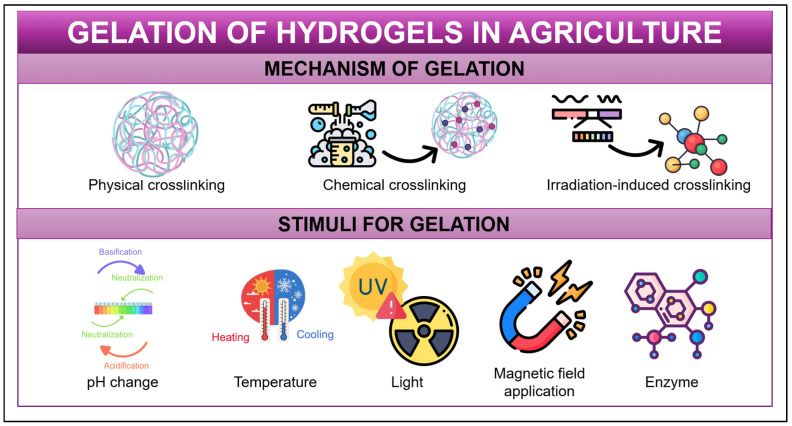
Mechanism of gelation and stimuli involved in the formation of hydrogels in agricultural applications.

The structural architecture of hydrogels is defined by interconnected polymeric networks, which are formed through the assembly of diverse polymer chains. Thus, gelation represents the process of polymeric networks forming in a solution of monomers or polymers [36,43,231]. This process may be further refined by utilizing different strategies, which include physical crosslinking, chemical crosslinking and irradiation-induced crosslinking techniques [281] (Figure 3).

In general, physical crosslinking occurs due to (i) hydrogen bonds, (ii) ionic interactions, (ii) hydrophobic interactions or crystallization, among others. These are weak interactions that can be changed by physical changes and do not promote modifications in chemical structure [6,282]. For instance, electrostatic interactions (also known as ionic interactions) can cause the stabilization of different networks [43,283]. This is evidenced by prior studies in which divalent ions (Ca^2+^) were added to alginate solutions [153] or trivalent ions (Fe^3+^) were employed in the formation of a hydrogel with carboxymethylcellulose [200].

In contrast, chemical crosslinking involves the formation of covalent bonds between polymer chains using different crosslinking reagents. For example, cellulose-based hydrogels can be prepared via physical or chemical crosslinking using compounds such as epichlorohydrin or citric acid [36,231,282,284]. Additional strategies for hydrogel network formation include different crosslinking strategies to reticulate different networks, such as the utilization of chemical elements as crosslinkers, like glutaraldehyde, for the preparation of gelatin [140], chitosan [136] or polylactic acid hydrogels, or the incorporation of epichlorohydrin in chitin solutions [125]. Another approach involves the addition of nanomaterials (e.g., montmorillonite, copper oxide nanoparticles, zein nanoparticles, cellulose nanofibers, among others) which allows the incorporation of these materials as additives (i.e., these materials do not prevent the incorporation of different crosslinkers) and promotes a modification of different properties (such as controlled agrochemical release, enhancement of mechanical properties or modification of absorption capacity) by the formation of different hydrogen bonds or van der Waals interactions [129,270,276,285]. An enzyme addition, such as transglutaminase, is also an alternative that can catalyze hydrogel crosslinking reactions; however, its application in agricultural hydrogels is limited due to stringent pH and temperature requirements [286].

Finally, an advanced technology is irradiation-induced crosslinking, which uses different sources of radiation (including gamma, electron bean or ultraviolet radiation), forming versatile materials [281].

However, there are different stimuli (Figure 3) that allow the formation of hydrogels such as temperature, electricity, light, magnetic field application, pH, enzymes, etc. [287]. The influence of temperature has been explored through the implementation of heating and cooling cycles, which promote the formation of non-covalent networks. For instance, gelatin is a compound that can form a hydrogel by decreasing the temperature by 5 °C, and similarly, drying–freezing cycles can be used to develop hydrogels using starch and cellulose as biopolymers [136,288]. Additionally, pH variations can trigger gelation in acid or base-induced gelation, as in the case of collagen and chitosan-based hydrogels, which undergo network formation upon the addition of NaOH; this system promotes the formation of a hydrogel network by linking hydrogen bonding [142,283]. Finally, ultraviolet and visible light, as well as irradiation, are widely used for the photoinitiation of molecules that absorb the incident radiation and trigger the formation of polymer networks. This approach has been primarily applied to synthetic polymers such as acrylic acid [89], vinyl polymers [109] and acrylamide [289], as shown in the literature.

## 4. Applications of Hydrogels in Agriculture

Among the main applications of hydrogels in agriculture, they are notable for their capacity to enable the controlled release of fertilizers, act as soil conditioners, improve water retention, and act as matrices for biostimulation and biofortification.

### 4.1. Controlled Release of Agrochemical Compounds

Firstly, one application of hydrogels in agriculture is the controlled release of agrochemical compounds, which needs to be synchronized with plant demand to optimize crop yield while reducing environmental contamination and providing economic benefits such as saving labor, time and energy. Particularly, the performance of CRFs is influenced by the presence of several hydrophilic groups, which can introduce functionality as well as factors including water solubility, microbiological decay, environmental temperature, particle size, and moisture content. Controlling these variables is essential for effective nutrient management, highlighting the differences in behavior in water compared to soil [290,291,292,293,294]. For instance, Supare et al. (2022) [137] developed chitosan–starch hydrogels that retarded the release of atrazine when compared with a commercial hydrogel, achieving a viability of 500 h. Table 6 summarizes selected studies on hydrogels incorporating fertilizers and herbicides. As observed, the main agrochemical studied is urea, which shows a longer controlled-release period when biopolymers such as starch or sodium alginate are employed, due to the possibility of crosslinking, thereby avoiding hazardous chemical emissions [295].

However, several mechanisms must be considered for the controlled release of agrochemical compounds. As described by Zanino et al. (2024) [301], the controlled release can be developed as a result of four mechanisms: (i) diffusion through water-filled pores, (ii) diffusion through the polymer network, (iii) matrix erosion and (iv) osmosis. Mainly, the first mechanism is typically found, and it is divided into three stages: (i) a lag phase, in which the agrochemical compound is released due to moisture entering the cavities; (ii) a steady-state phase, which occurs due to the swollen hydrogel from the diffusion of the concentration of the solution, promoting an immediate release; and (iii) a degradation stage, during which most of the compounds have been released [293,302,303]. This process can be influenced by multiple factors, such as the thickness of the material’s membrane or its water solubility. However, osmosis processes must also be considered, as they play a role in heterogeneous matrices such as soils, where water absorption is modified due to pressure that promotes pore expansion [278]. Optimizing the release time in relation to the time needed for the plant is necessary, which hinders the manufacturing process due to the broad number of possible plants and soils [304].

In any case, materials must possess good biodegradability to mitigate the contamination of soils by the formation of products such as organic matter, carbon dioxide and water [36]. This is important for petrochemical-based materials and synthetic polymers, which can form toxic products as well as microplastics; it is necessary to control polymerization to produce lower molecular weight hydrogels with reduced environmental impact [74]. On the other hand, natural polymers are decomposed by soil enzymes or microorganisms, resulting in structural changes [117]. The degradation rate is influenced by different factors, including pH, temperature, oxygen content, humidity, the presence of enzymes, heat, sunlight or the availability of nutrients [36,305]. Therefore, it is necessary to evaluate the long-term accumulation of these types of materials in the soil, aiming to reduce their ecotoxicity and health risks, due to the existence of multiple studies at the lab scale, as displayed in Table 7.

### 4.2. Soil Conditioner and Water Retainer

Another typical application of this type of matrix is as a soil conditioner, due to its ability to mitigate the effects of salinity while promoting plant growth and maintaining soil structure. Specifically, hydrogels are materials that improve porosity, density, structure, permeability and aeration, reducing compaction and improving microbial activity, which are essential for mitigating the degradation of soils caused by conventional fertilizers [308,309,310]. Therefore, these materials have an important impact on plant growth, as shown in the studies displayed in Table 8, which particularly focus on the increase in root weight, number of fruits, extension of the growth cycle, etc. However, as observed, these types of analyses are mainly developed under laboratory conditions, making their translation to real crops necessary to ensure the correct behavior for transitioning from conventional soil conditions to hydrogels [295].

This property is generally related to their ability to act as water retainers, a property that is crucial for ensuring water conservation in agricultural activities under drought conditions. Their water retention capacity, however, can be modified due to the multitude of soil characteristics, including hydraulic properties, aggregate stability and textures. In this context, researchers generally evaluate their swelling capacity (Table 9), which is influenced by a multitude of factors, such as pH and ionic strength. Qin et al. (2022) [311] synthesized cellulose-based hydrogels that functioned as soil amendments, increasing water uptake in wheat plants by 94.7% and improving the average number of leaves in comparison to potassium polyacrylate.

**Table 8:** Effect of hydrogels on plant growth, including information about the main polymer used.

Main Polymer	Effect on Plant Growth	Reference
Cellulose and polylactic acid	Enhanced the growth and survival rate of Raphanus sativus and Phaseolus vulgaris by 20%	[300]
Starch, polyacrylic acid, polyvinyl alcohol	Improvement in chili plant growth	[110]
Carboxymethyl cellulose	More significant cucumber seed germination	[186]
Carboxymethyl cellulose, hydroxyethyl cellulose	Hydrogels tripled the time during which the soil remained humid	[312]
Gum arabic, acrylic acid	A 20% increase in the diameter and volume of roots in maize plants	[279]
Carboxymethyl cellulose, acrylic acid	Hydrogels promoted plant growth and germination in 28 days	[88]
Gelatin, methacrylamide, agar	Enhancement of the retention of water in chickpea plants	[165]
Agar, activated carbon	Plants with hydrogels showed better and more uniform growth than those without hydrogels	[164]
Chitosan, polyvinyl alcohol	The implementation of hydrogels increased the stem length and diameter by approximately 20% in watermelon	[313]
Sodium alginate, lignosulfonate, konjac	Under extreme conditions, hydrogels were able to extend the growth cycle of the plant by 9–14 days	[151]
Chitin	Seeds germinated very quickly when using soilless culture media	[125]
Sodium alginate	Hydrogels promoted the growth of maize seedlings by continuously providing water and nutrients that the plant needs	[157]

### 4.3. Enhancers of the Soil Biological Activity

Finally, these materials can also be applied for biostimulation purposes because of their ability to stimulate soil biological activity [314,315]. For instance, de Carvalho et al. (2024) [316] developed a gelatin-based hydrogel coated with urea, which exhibited a notable effect on lettuce seeds, resulting in significant improvements in germination and plant growth. Similarly, Nuzzo et al. (2020) [317] reported the development of hydrogels incorporating different humic-like substances, highlighting their potential for direct use as plant biostimulants. As a matter of fact, hydrogels can also be utilized for crop biofortification, which is based on enriching food with essential dietary micronutrients. For instance, Afnan et al. (2024) [318] reported the use of hydrogels to enhance the availability of vitamin C. Similarly, Mikula et al. (2020, 2024) [319,320] developed different biobased hydrogels containing zinc, manganese and copper as micronutrients, promising their application for the development of biofortified foods through the generation of sprouts enriched with these elements.

**Table 9:** Water swelling capacity (WSC) of different hydrogels, including information on the main polymer used and the conditions of the experiment.

Main Polymer	Conditions	WSC (g/g)	Reference
Sodium carboxymethyl cellulose, hydroxyethyl cellulose, polyvinyl alcohol	Distilled water at a media temperature between 10 and 40 °C	5.00–18.40	[321]
Xanthan gum, gelatin	Distilled water at 25 °C	9.80–25.00	[202]
Gelatin, chitosan, polyvinyl alcohol	Distilled water	10.80–12.00	[215]
Lignin	Distilled water	34.00	[197]
Alginate	Deionized water	3.09	[322]
Cellulose, carboxymethyl cellulose	Distilled water at 26 °C	14.50–20.55	[194]
Chitosan	Distilled water	2.49–3.25	[323]
Starch, poly(vinyl alcohol)	pH 7 and 25 °C	5.0–12.00	[324]
Carboxymethyl cellulose, polyvinylpyrrolidone	Distilled water at room temperature	130.00–144.00	[325]
Carboxymethyl cellulose, hydroxyethyl cellulose	Distilled water	30.00–80.00	[312]
Chitosan	pH 1 at room temperature	0.80–0.90	[136]
Chitosan, gelatin, polylactic acid	Deionized water at 25 °C	44.48	[140]

## 5. Challenges

Hydrogels have gained special interest within the methods employed in sustainable agriculture due to a multitude of their characteristics compared to other conventional materials. Thus, it is possible to distinguish different advantages in their use, such as:

- Their water absorption capacity is much higher than that of other organic mulches, charcoal or other conventional plastic mulches [51].
- Intelligent systems capable of responding to stimuli highlight their ability to modulate the delivery of such nutrients compared to conventional fertilizers or those with controlled release [295].
- Soil properties and microbial activity improve, since they are environmentally friendly, provide a fixed content of organic matter and lack of pathogens compared to organic amendments or the carbon trapping caused by charcoal [74].

However, despite their multiple advantages, hydrogels present several challenges that researchers must address.

- Firstly, it is necessary to optimize the appropriate amount of polymer, aiming to achieve an adequate degree of crosslinking while avoiding negative effects. This concentration will vary depending on the type of biopolymer as well as its chemical properties (e.g., molecular weight, viscosity, among others). Nonetheless, the combination of different formulations must be considered to enhance the synergistic effects of the compounds, such as the combination of polysaccharides with proteins as a source of nitrogen. Likewise, the incorporation of different crosslinkers for the development of chemically and enzymatically crosslinked hydrogels must be considered to avoid toxicity and potential harm to soils [326].
- Moreover, the key properties of hydrogels that enable them to act as water reservoirs are their ability to retain water and saline solutions. Therefore, it is necessary to measure these properties and their release into soils, particularly given the ability of cryogels (which are a special type of hydrogel) to undergo multiple swelling–shrinking cycles under different external stimuli. Another alternative is their utilization as matrices for the controlled release of agrochemical compounds, whose incorporation must be characterized to ensure a proper response, allowing their release in soil and being directly linked to the characteristics of the hydrogel. Importantly, release profiles should align with peak plant nutrient demands to prevent leaching or depletion [327,328,329].
- With respect to their incorporation into soils, it is necessary to evaluate the properties of soils before and after the incorporation of the matrices, aiming to avoid pollution due to various possible causes (acidification or alkalinization of soils, which can alter the availability of macronutrients and micronutrients; formation of carbonates; and a decrease in organic matter content, which worsens due to the overuse of chemical fertilizers and which needs to be maintained in equilibrium to support soil development, etc.). Nonetheless, biodegradability into non-toxic products must be achieved to prevent environmental contamination, as well as characterization of the effects of microorganisms present in soils [36,43,330].
- It is also essential to evaluate the effects of hydrogels on plants. Although the incorporation of a porous gelled matrix into soils will not be detrimental to plant growth, several factors must be considered to ensure agricultural production. Thus, the germination rate, number of leaves, total biomass, presence of different nutrients in the fruits and overall crop yield must be evaluated. Additionally, toxicity tests are required to prevent potential risks to human health [331,332].
- Moreover, the stability of micronutrients in the soil, their absorption by roots, interactions with other nutrients, and possible losses due to leaching are essential to evaluate for biofortification applications [333,334].
- Industrially, a cost–benefit balance is essential. Hydrogels can be produced from treated residues and by-products from agricultural, industrial and marine sources, which can be treated to obtain different biopolymers [335,336,337]. However, their cost is often prohibitive for smallholder farmers in comparison with other traditional methods, such as mulching or composting [62], highlighting the need for scalable methods for their manufacture [338].
- It is necessary to take into consideration the existence of laws that ensure safe use of biopolymers and nanomaterials; particularly in Europe, the main regulating laws are Regulation (EU) 2019/1009 and REACH for chemical substances, while EFSA develops specific guidelines for assessing the risks of nanomaterials in the food chain. Hence, these regulations limit the use of hydrogels by evaluating their toxicity, biodegradability, ecotoxicity, as well as persistence in soils [339].

## 6. Conclusions and Future Perspectives

This review contextualizes the state-of-the-art of hydrogels applied in agriculture, which has undergone significant advancements in recent years. Current research primarily focuses on developing biopolymer-based hydrogels that exhibit unique properties due to their non-toxic, biocompatible, and biodegradable nature, thereby highlighting the potential for promoting a circular economy in which agri-food waste and by-products can find new applications. Moreover, the combination of these biopolymers with other established compounds is being explored to enhance their intrinsic characteristics and reduce reliance on synthetic polymers, whose biodegradation rate is relatively slow. Numerous studies have demonstrated that hydrogels have the potential to serve as effective carriers for nutrients and water, thereby promoting plant growth in drought conditions or enhancing soil quality.

However, there are still some challenges with the development of these multifaceted materials that scientists should focus on:

- Hydrogel composition and performance. The composition of biopolymer-based hydrogels must be carefully evaluated to avoid negative impacts. Therefore, because of their ability to be modified by external factors (pH, temperature or ion concentration), it is necessary to assess the changes in their efficiency under unfavorable conditions (high temperatures, soil compaction or salinization). This includes in-depth studies of the mechanical properties of the hydrogels to optimize their water retention capacity while maintaining ease of handling and application.
- Soil compatibility. The survival of soil microorganisms must be ensured, requiring a thorough evaluation of hydrogel–soil interactions by studying multiple characteristics such as soil texture, soil composition, biodegradation rates and changes in microbial populations to ensure compatibility and environmental safety.
- Nutrient release. Nutrient release has been widely studied for macronutrients (such as nitrogen and phosphorus), but it must be expanded to include micronutrients (like zinc, copper, etc.), particularly those that are deficient in the population, to encourage crop biofortification strategies.
- Plant response optimization. The effectiveness of hydrogels must be assessed through parameters such as germination rate, root and shoot length, biomass, crop yield or seed composition. The need for hydrogels depends on the specific requirements of each plant species.
- Industrial considerations. Scaling up hydrogel production is necessary to optimize production processes, reduce costs and improve scalability, thereby enabling practical and economically viable field applications.

In conclusion, the synthesis and application of hydrogels for agricultural purposes remain a dynamic and challenging area of research, necessitating significant advancements in several areas, including the development of innovative strategies for creating superabsorbent materials to address water scarcity, the use of protein as raw material and primary source of nitrogen for fertilization, the integration of nanomaterials to enhance the mechanical and functional properties of the hydrogels and the establishment of in vivo studies to evaluate their impact on plant growth. Additionally, standardized testing protocols for agricultural hydrogels, long-term field trials, cost analyses, farmer adoption, and regulatory harmonization across regions are needed. Furthermore, expanding hydrogel applications to biofortified crops could play a critical role in combating malnutrition and improving global food security. With continued innovation and a commitment to sustainable development, hydrogels represent a transformative tool for sustainable agriculture, capable of enhancing water efficiency, nutrient delivery, and crop resilience, while contributing to a circular bioeconomy and global food security.

## Figures and Tables

**Table 1:** List of commercially available hydrogel products used in agriculture, including the raw materials, key properties and manufacturers.

Raw Materials	Commercial Hydrogel	Properties	Manufacturer	References
Potassium polyacrylate (KPA)	Plant Hydrogel; Potassium Polyacrylate	Absorbs 300 times its weight in water Enhances seed germination and root development Increases soil water retention Enhances the utilization of organic matter within the soil Decreases the loss of fertilizers	Alquera Ciencia SL (Spain)	[63]
	TRPSORB^TM^	High water absorption capacity and controlled release of nutrients Long life cycle (1–3 years) Significantly increases seedling survival rate	Qingdao SOCO New Material Co., Ltd.	[64]
	Control Garden	Optimizes water use Suitable for hot climates Facilitates the growth of roots	GrowMania SL (Spain)	[58]
	Plara	Increases plant drought resistance Ensures a stable water supply for plants Improves the quality and size of fruits and vegetables	Plara Sp.z.o.o (Poland)	[59]
	ALSTA Hydrogel	Ability to absorb up to 500 times its weight in water Compatible with all soil and plant types	Chemtex SL (India)	[65]
Potassium-based polymer	Agua Gel	Biodegradable Decrease water irrigation cycles Increase soil porosity	Novingrecons SLU (Spain)	[66]
Hydrolyzed gelatin	Agrogel	Enables complexation of macronutrients Controlled release of nitrogen via microbial activity Ability to absorb between 150–200% of deionized water	ILSA S.p.A (Italy)	[67]
Carboxymethyl cellulose-grafted anionic polyacrylate polymer	Pusa Hydrogel	High fluid absorption in the presence of fertilizers Maintains water absorption capacity at high temperatures Slow release of water Employment for an extended time	IARI, New Delhi	[68]
Acrylate/Acrylamide copolymer	Waterlock 93N	Absorbs 100 times the water during multiple cycles Moisture environment Biodegrades into CO_2_, H_2_O, nitrogen, etc. Can be used for 2–3 years	Acuro Organics Ltd., New Delhi	[69]

**Table 2:** Raw materials for hydrogels applied to agriculture depend on their origin, including synthetic polymers and natural polymers.

**RAW MATERIALS** **FOR HYDROGELS IN AGRICULTURE**	SYNTHETIC POLYMERS	Acrylic acid and acrylic derivatives	
		Vinyl polymers	
		Polyethylene glycol and polyurethanes	
	NATURAL POLYMERS	Based on polysaccharides	Chitin and chitosan
			Sodium alginate
			Agar
			K-carrageenan
			Starch
			Cellulose
			Lignin
			Xanthan gum
			Guar gum
		Based on proteins	Collagen and gelatin
			Keratin
			Soy protein

**Table 3:** Major advantages and disadvantages of synthetic polymer-based and natural polymer-based hydrogels.

Classification of Hydrogels	Advantages	Disadvantages
Synthetic polymer hydrogels	High water absorption capacity Durability Tunable mechanical properties Tunable swelling properties Effectiveness under drought conditions	Poor biodegradability Possible presence of residual monomers Potential toxicity
Natural polymer hydrogels	Biodegradability Environmentally friendly Derived from renewable resources Non-toxic	Lower water absorption capacity Rapid degradation in soil Weak mechanical properties

**Table 6:** Hydrogels for controlled release of agrochemical products, including information on the main polymer used, the agrochemical, and the release time.

Main Polymer	Agrochemical (AC)	Release Time	Reference
Sodium alginate, acrylic acid, acrylamide	Urea	100% within 20 days	[97]
Gelatin, polyacrylamide	Urea	100% within 6 days	[296]
	P_2_O_5_		
	K_2_SO_4_		
Agar, Starch	Atrazine	100% after 6 days	[297]
Gelatin, methacrylamide, agar	Linuron	100% after 3 days	[165]
Sodium alginate, carboxymethyl chitosan	Urea	75–90% after 26 days	[139]
Sodium alginate, starch	Diammonium hydrogen phosphate	10–12 wt% after 30 days	[298]
Halloysite, sodium alginate	Urea	100% after 36–60 h	[299]
Bentonite, sodium carboxymethyl cellulose	Metolachlor	50% after 158 h	[181]
Starch, chitosan	Atrazine	750 mg/0.5 g hydrogel after 750 h	[137]
Starch	Urea	80–90% after 42 h	[267]
Starch	Urea	80–90% after 30 days	[176]
Sodium alginate, starch	Urea	100% after more than 50 days	[157]
Cellulose, polylactic acid	Potassium nitrate	85% within 96 h	[300]
Starch, polyacrylic acid, polyvinyl alcohol	Urea	35–75% within 30 days	[110]
Carboxymethyl cellulose, acrylic acid	Urea	210–220 mg/dL hydrogel for 144 h	[86]
Carboxymethyl cellulose	NPK fertilizer	72 h	[186]
Carboxymethyl cellulose, polyacrylic acid	Phosphorus	27–32% after 150 min	[88]

**Table 7:** Biodegradability of different hydrogels, including information about the main polymer used, the conditions and the days for biodegradation.

Main Polymer	Conditions	Range of Days of Biodegradation	Reference
Sodium alginate, carboxymethyl cellulose	Room temperature for 30 days	21.4–25.5% of degradation after 30 days	[139]
Sodium alginate, chitosan	Room temperature for 30 days	30.2716–33.5697% of degradation after 30 days	[141]
Lignin and PEDGE	Room temperature for 40 days	15–82% of degradation for 40 days	[197]
Xanthan gum, gelatin	Room temperature for 40 days	65–70% mass loss after 60 days	[202]
Soy protein isolate, polyvinyl alcohol	Room temperature for 100 days	65.18% mass loss after 100 days	[220]
Gelatin, polyvinyl alcohol	Room temperature for 28 days	40–47% mass loss after 28 days	[215]
Sodium alginate, polyvinyl alcohol	Room temperature for 120 days	36.9–47.4% weight loss after 120 days	[149]
Acrylic acid	30 °C for 60 days	63% weight loss after 60 days	[306]
Guar gum, pectin, acrylamide	For 30 days	45% of the mass was degraded after 30 days	[307]
Sodium alginate	Room temperature for 60 days	6% of the mass was in the first 60 days and 14% after the second 60 days	[151]

## Data Availability

No data were generated during this review article.
